# 

**Published:** 1915-05-22

**Authors:** 


### CONTENTS.

PAGE

**The Provision for Cases of Senile Insanity** ... 159

**A War Emergency Committee for London** ... 160

**Hospital and Institutional News** ... ... ... 161

Medical Prisoners of War—Visits to the Wounded—Hospitals and the Spirit Duty—Sir Edgar Speyer and the King's Fund—The Value of the Fellowship—Is a "Winkle" a Fish?—Shells as Prophylactics—What is a Still-Born Child?—Lister's House in London—Conference on the Feeble-Minded—The Belvidere Hospital Inquiry—Strikers and the Wounded—A Medico-Legal Point—The Navy and Military Doctors—The Lister Institute—Medical Recruits—Medical Officer and His Half-Pay—A Medical Officer's Bravery—Poor-Law Medical Officers' Association and Lady Visitors—New Medical Association in Ireland—Doctors and Dentistry—The War and the Cost of Institutional Treatment—Mental Superintendent's Resignation—Mental Treatment Without Certification—Face-Piece for Poisonous Gases—Pensioners and Medical Assistance—Drugs and Optic Atrophy—Italy and the Drug Market.

**London and Counties Medical Protection Society** ... ... ... ... ... ... 167

The Profession and its Safeguards.

**Mumps and Parotitis** ... ... ... ... 168

**Disease Germs and Evolution** ... ... ... 169

Epidemics, War, and the Public Health.

**Hospital and Health Law in the Empire and U.S.A.** ... ... ... ... ... ... ... 170

**A Rural Tuberculosis Dispensary** ... ... 171

A Guide to its Equipment and Cost.

**Medical Appointments** ... ... ... ... 172

**Round the World** ... ... ... ... ... 173

Fellow-Workers and the Roll of Honour.

*[See over.]*

**JAMES SLATER & CO. (Engineers) LTD.**

**50 & 51 WELLS STREET, W.**

**HEATING, HOT WATER SUPPLY, VENTILATION, ETC.**

We make a great speciality of this, and are at present engaged upon the entire installation at the new King's College Hospital, including central steam boiler plant, etc., etc.

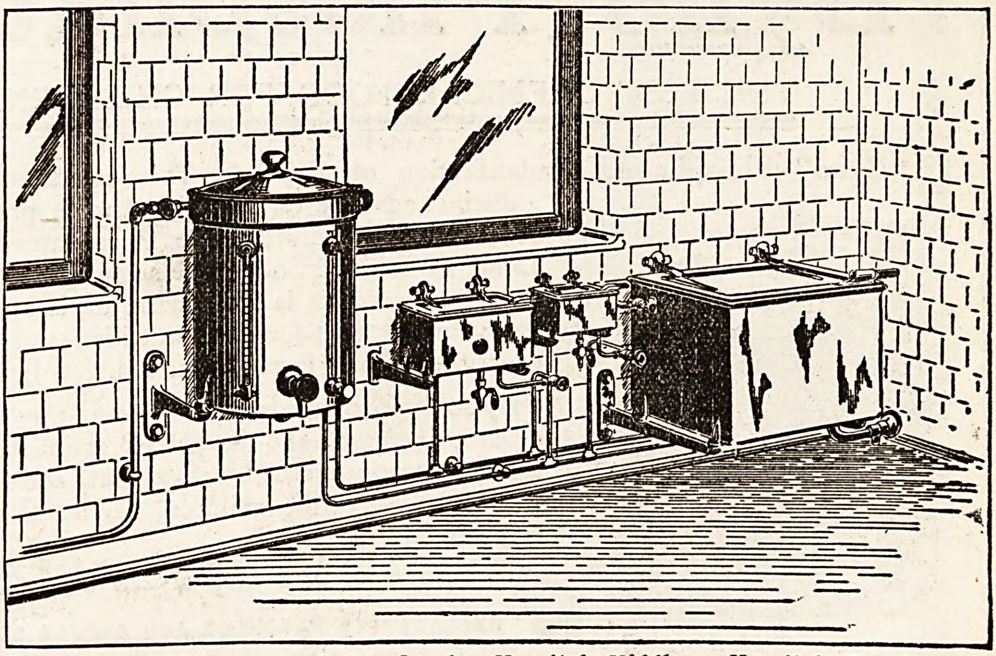

**STERILIZERS, as supplied to London Hospital, Middlesex Hospital, Manchester Royal Infirmary, etc.**

### CONTENTS—(*continued*).

| | PAGE |
|---|---|
| **How to Sign a Prescription** ... ... ... | 174 |
| The Latest Insurance Act Problem. | |
| **The Editor's Letter-Box** ... ... ... ... | 175 |
| Fires Due to Air Raids. | |
| **London Panel Committee** ... ... ... ... | 175 |
| The Enlistment of Insured Persons. | |
| **The Department of Modern Nursing** ... ... | 176 |
| XIII.—The Gloucestershire Royal Infirmary and Eye Institution. | |
| **Order of St. John** ... ... ... ... ... | 176 |
| **The Training of Soldier-Nurses** ... ... ... | vi |
| Field Ambulances and their Working: By Josiah Oldfield, M.R.C.S., L.R.C.P. | |
| **Personalia** ... ... ... ... ... ... | vi |
| **Gifts and Bequests** ... ... ... ... ... | viii |
| **Institutional Needs** ... ... ... ... ... | viii |
| **Doctors' Wills** ... ... ... ... ... ... ... | viii |
| **Buildings Contemplated and in Progress** ... | viii |
| **The Book Market** ... ... ... ... ... | viii |
| **The Institutional Worker** ... ... (Suppl.) | 1 |
| VIII.—The Junior Clerk. | |
| Question for May. | |
| Index System for Minute-Books: Answer to April Question. | |
| Enquiries and Answers. | |
| The Sick and in Need: Nurse Suffering from Rheumatism. | |
| Employment and Training: Training at Thirty-Nine—Hospital Training—Post-Graduate Experiences—Training in Nervous Diseases—Names of Hospitals and Handbook of Technical Terms. | |

